# Indoxyl sulfate caused behavioral abnormality and neurodegeneration in mice with unilateral nephrectomy

**DOI:** 10.18632/aging.202523

**Published:** 2021-02-17

**Authors:** Chiao-Yin Sun, Jian-Ri Li, Ya-Yu Wang, Shih-Yi Lin, Yen-Chuan Ou, Cheng-Jui Lin, Jiaan-Der Wang, Su-Lan Liao, Chun-Jung Chen

**Affiliations:** 1 Department of Nephrology, Chang Gung Memorial Hospital, Keelung 204, Taiwan; 2 Community Medicine Research Center, Chang Gung Memorial Hospital, Keelung 204, Taiwan; 3 Kidney Research Center, Chang Gung Memorial Hospital, Taoyuan 333, Taiwan; 4 School of Medicine, Chang Gung University, Taoyuan 333, Taiwan; 5 Division of Urology, Taichung Veterans General Hospital, Taichung 407, Taiwan; 6 Department of Family Medicine, Taichung Veterans General Hospital, Taichung 407, Taiwan; 7 Institute of Clinical Medicine, National Yang Ming University, Taipei 112, Taiwan; 8 Center for Geriatrics and Gerontology, Taichung Veterans General Hospital, Taichung 407, Taiwan; 9 Department of Urology, Tungs’ Taichung MetroHarbor Hospital, Taichung 435, Taiwan; 10 Division of Nephrology, Department of Internal Medicine, Mackay Memorial Hospital, Taipei 104, Taiwan; 11 Mackay Junior College of Medicine, Nursing and Management, Taipei 251, Taiwan; 12 Children’s Medical Center, Taichung Veterans General Hospital, Taichung 407, Taiwan; 13 Department of Industrial Engineering and Enterprise Information, Tunghai University, Taichung 407, Taiwan; 14 Department of Medical Research, Taichung Veterans General Hospital, Taichung 407, Taiwan; 15 Department of Medical Laboratory Science and Biotechnology, China Medical University, Taichung 404, Taiwan; 16 Ph.D. Program in Translational Medicine, College of Life Sciences, National Chung Hsing University, Taichung 402, Taiwan

**Keywords:** depression, gut microbiota, neurodegeneration, neuroinflammation, uremic toxin

## Abstract

Chronic Kidney Disease (CKD) and neurodegenerative diseases are aging-related diseases. CKD with declined renal function is associated with an elevation of circulating indoxyl sulfate, a metabolite synthesized by gut microbes. We explored the roles of gut microbial metabolites in linking with Central Nervous System (CNS) diseases by administrating indoxyl sulfate intraperitoneally to male C57BL/6 mice with unilateral nephrectomy. Upon exposure, the accumulation of indoxyl sulfate was noted in the blood, prefrontal cortical tissues, and cerebrospinal fluid. Mice showed behavioral signs of mood disorders and neurodegeneration such as anxiety, depression, and cognitive impairment. Those behavioral changes were accompanied by disturbed neuronal survival, neural stem cell activity, expression of Brain-Derived Neurotrophic Factor, serotonin, corticosterone, and Repressor Element-1 Silencing Transcription Factor, and post-receptor intracellular signaling, as well as upregulated oxidative stress and neuroinflammation. Uremic toxin adsorbent AST-120 improved the above mentioned changes. Intriguingly, intracerebroventricular indoxyl sulfate administration only caused limited alterations in the normal mice and the alterations were reversed by aryl hydrocarbon receptor antagonism. The findings suggest pathogenic roles of indoxyl sulfate in the development of CNS diseases, and highlight gut microbiota as alternative targets for intervention with the aim of slowing down the progression of CKD and decreasing CNS complications.

## INTRODUCTION

The gut microbiota is pivotal to human health and diseases. Homeostasis is maintained in the gut by the diverse microbial community in the gut microbiota, which is known to have crucial roles in the host’s physiological processes. Aging-associated changes in the gut microbiota resemble those associated with dysbiosis. Low diversity in gut microbiota is closely linked with aging and predisposes susceptible individuals to a number of diseases [1, 2]. Transfer of aged gut microbiota confers pro-inflammatory phenotype in young germ-free mice [3], suggesting a substantial role of gut microbiota in disease initiation and progression. Aging and dysbiosis share inflammation as a common hallmark and inflammation is implicated in the progression of age-related diseases [4]. Therefore, there has been considerable interest in exploring therapeutic options aimed at restoring the gut microbiota in order to promote health and treat disease, particularly aging-related diseases.

The gastrointestinal tract and the Central Nervous System (CNS) have been shown to communicate bidirectionally through the gut-brain axis. Accumulating evidence indicates that gut microorganisms are closely associated with various CNS diseases, such as Parkinson's disease, Alzheimer's disease, schizophrenia, and multiple sclerosis [5, 6]. Abnormalities in the microbiota-gut-brain axis have also been highlighted as an emerging component in the pathogenesis of depression. Clinical findings reveal that recurrent exposure to antibiotics was associated with increased risk for depression, while use of probiotics/prebiotics appeared to be protective against depression [7, 8]. Rodent studies of fecal microbiota transplantation, probiotic consumption, and fluoxetine antidepressant treatment further suggest a critical role of gut microbiota in the pathogenesis of depression [9–11]. However, the precise mechanisms underlying the interaction between the CNS and the gastrointestinal system with respect to mood disorders and neurodegeneration remain unclear.

Although a disturbed gut microbiota has been implicated in a number of CNS diseases, its pathogenic mechanisms and surrogates have yet to be determined. Disturbed gut microbiota causes an elevation of circulating indoxyl sulfate and p-cresol sulfate, bioactive metabolites of tryptophan and tyrosine, respectively, which are produced by gut microbiota [12]. Higher cerebrospinal fluid (CSF) accumulation of these metabolites has been observed in patients with Parkinson’s disease [13]. Furthermore, indoxyl sulfate displays pro-inflammatory effects by acting on CNS glial cells [14, 15]. Since neuroinflammation contributes substantially to most CNS diseases, indoxyl sulfate is theoretically a microbiota-derived metabolic surrogate for the development of CNS diseases.

The varied functions of the kidneys are influenced by the complex processes of aging and superimposed diseases. The risk of Chronic Kidney Disease (CKD) is high in the elderly and plasma levels of uremic toxins increase with age [16, 17]. Indoxyl sulfate, a well-known protein-bound uremic toxin, is a pathogenic factor of CKD and cardiovascular diseases [18]. Renal impairment is associated with accumulation of uremic toxins in the brain and CNS dysfunctions [19, 20]. Clinically, depression is highly prevalent among patients with CKD [21]. Thus, there appears to be a pathological linkage between indoxyl sulfate and CNS diseases, particularly depression. Unlike severe experimental CKD models by adenine feeding or 5/6 nephrectomy, rodent models with CKD by unilateral nephrectomy present the normal renal function, while showing biological activities of exogenous uremic toxins [22–24]. Alteration in the gut microbiota has been demonstrated in patients with CKD [25]. To further explore the role of gut microbial metabolites in CNS diseases, a mouse model with unilateral nephrectomy was established in which indoxyl sulfate was administered daily. The possible pathological role of indoxyl sulfate was investigated by monitoring behavioral changes in the mice and by determining the molecular mechanisms involved.

## RESULTS

### Indoxyl sulfate caused behavioral changes

**Figure 1:**
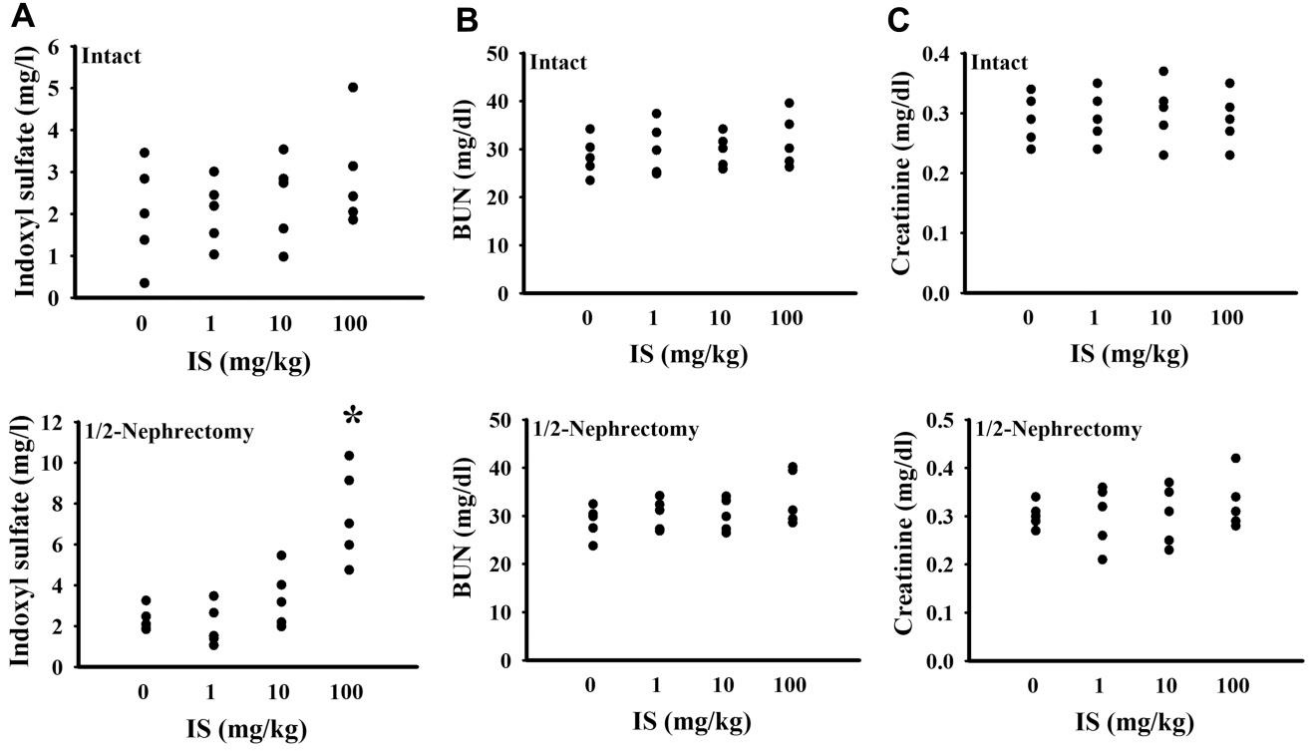
**Indoxyl sulfate administration caused indoxyl sulfate serum accumulation in unilateral nephrectomized mice.** The intact and unilateral nephrectomized (1/2-Nephrectomy) mice were intraperitoneally injected with various doses of indoxyl sulfate (IS) for 7 weeks. The blood was collected and subjected to the measurement of indoxyl sulfate (**A**), BUN (**B**), and creatinine (**C**). *p < 0.05 vs. intact control group without indoxyl sulfate, n = 5.

**Figure 2:**
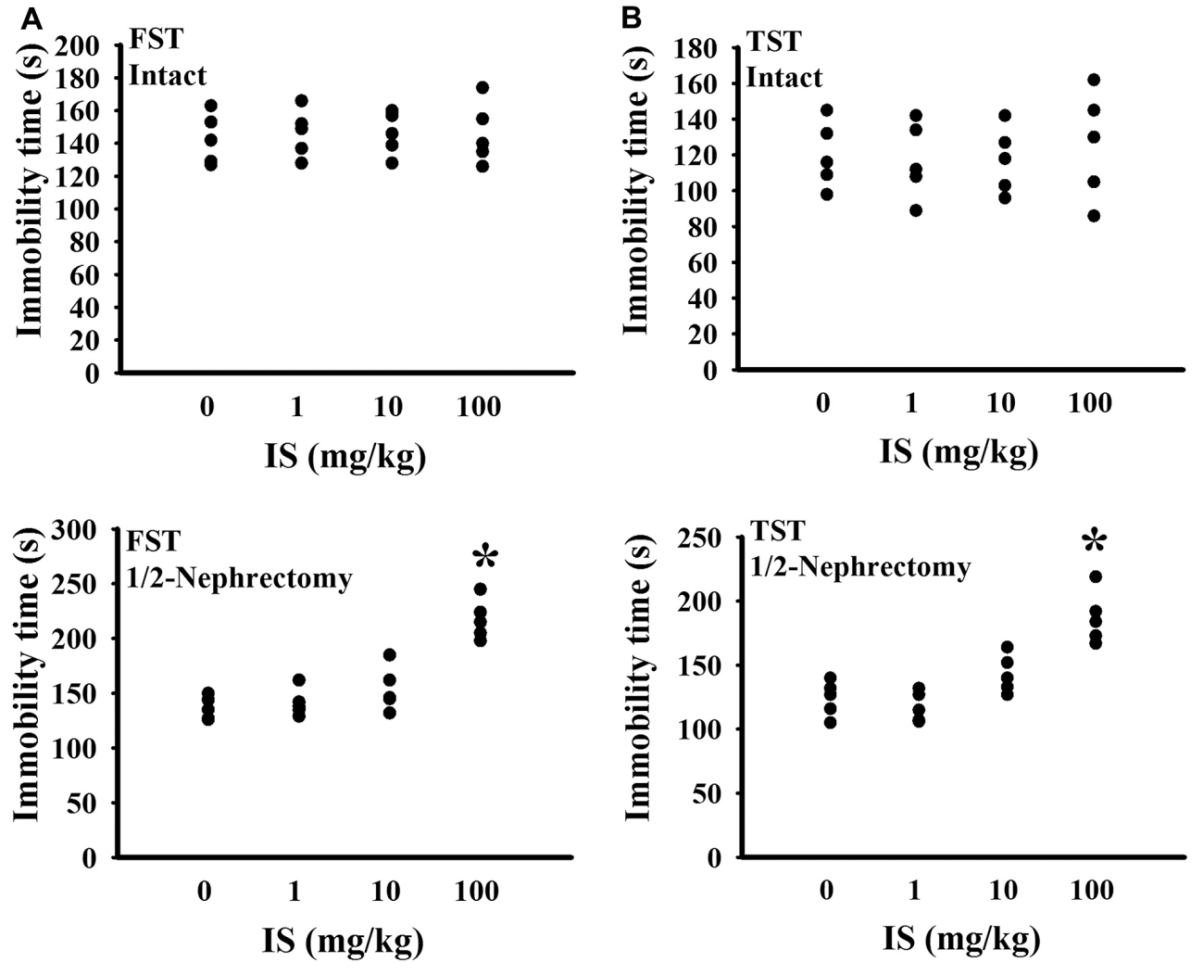
**Indoxyl sulfate caused alterations in unilateral nephrectomized mice.** The intact and unilateral nephrectomized (1/2-Nephrectomy) mice were intraperitoneally injected with various doses of indoxyl sulfate (IS) for 7 weeks. A FST was conducted for a period of 5 minutes and the duration of immobility was recorded (**A**). A TST was performed for a period of 6 minutes and the duration of immobility was recorded (**B**). *p < 0.05 vs. intact control group without indoxyl sulfate, n = 5.

To find out potential effects of indoxyl sulfate, consequences of various doses (0, 1, 10, and 100 mg/kg) were primarily explored in mice with intact or unilateral nephrectomy. Only high doses at 100 mg/kg, unilateral nephrectomized mice elevated indoxyl sulfate level in blood circulation (p < 0.05, Figure 1A), while presented normal ranges of plasma Blood Urea Nitrogen (BUN) (p > 0.05, Figure 1B) and creatinine (p > 0.05, Figure 1C). Additionally, high dose indoxyl sulfate-exposed mice (100 mg/kg) showed an increased immobility time in the Forced Swimming Test (FST) (p < 0.05, Figure 2A) and Tail Suspension Test (TST) (p < 0.05, Figure 2B), implying potential CNS effects. Intriguingly, the effects of indoxyl sulfate were not duplicated in kidney intact mice (Figures 1, 2). Therefore, unilateral nephrectomized mice and indoxyl sulfate at dose of 100 mg/kg were utilized throughout all subsequent experiments.

**Figure 3:**
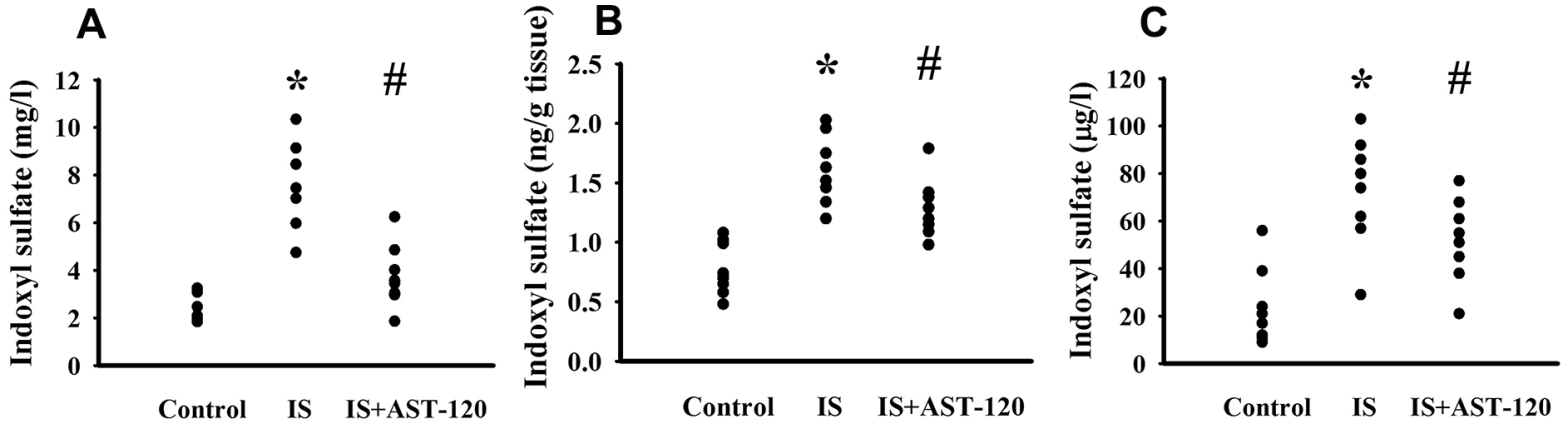
**Peripheral indoxyl sulfate treatment increased its blood and CNS distribution.** Unilateral nephrectomized mice were intraperitoneally injected with indoxyl sulfate (IS, 0 and 100 mg/kg) and the indoxyl sulfate-injected mice were orally given with AST-120 (0 and 400 mg/kg) for 7 weeks. The blood (**A**), prefrontal cortical tissue (**B**), and CSF (**C**) were isolated and subjected to the measurement of indoxyl sulfate. *p < 0.05 vs. control group, and #p < 0.05 vs. indoxyl sulfate alone (IS) group, n = 8.

**Figure 4:**
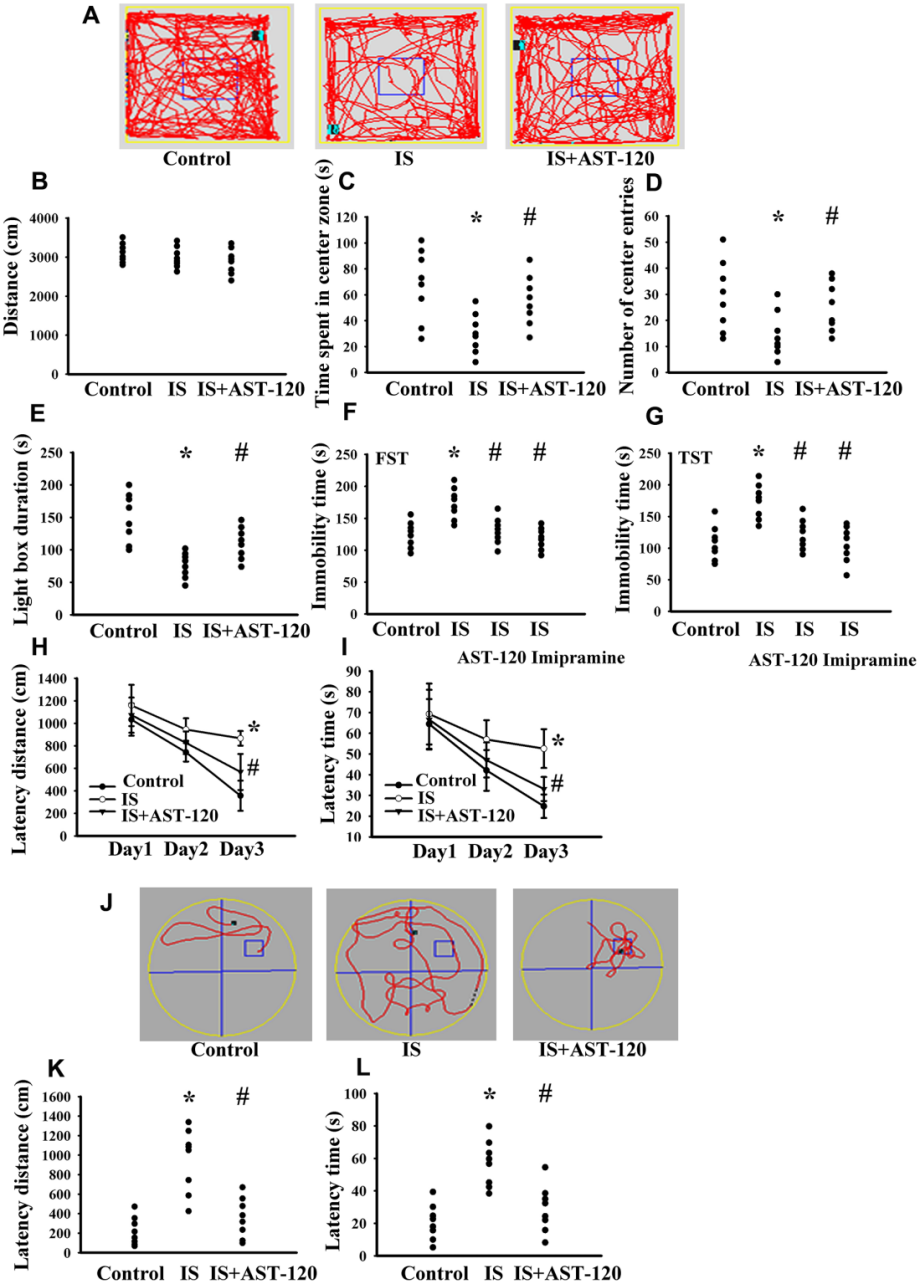
**Indoxyl sulfate caused behavioral alterations**. Unilateral nephrectomized mice were intraperitoneally injected with indoxyl sulfate (IS, 0 and 100 mg/kg) and the indoxyl sulfate-injected mice were orally given with AST-120 (0 and 400 mg/kg) for 7 weeks. The tracking paths (**A**), distance in movement of spontaneous locomotor activity (**B**), time spent in the center zone (**C**), and numbers of center zone entries (**D**) were evaluated by the Open Field Test. The duration of light preference was evaluated by the Light-Dark Box Test (**E**). The FST was conducted for a period of 5 min and the duration of immobility was recorded (**F**). The TST was performed for a period of 6 min and the duration of immobility was recorded (**G**). Antidepressant imipramine (20 mg/kg) was intraperitoneally administrated 1 h prior to the FST (**F**) and TST (**G**). In the Morris Water Maze Test, the escape distance (**H**) and escape time (**I**) in the acquisition phase were recorded from 1^st^ to 3^rd^ days. After training for 3 consecutive days, the swimming routes (**J**), escape distance (**K**), and escape time (**L**) required to reach the hidden platform were recorded. *p < 0.05 vs. control group, and #p < 0.05 vs. indoxyl sulfate alone (IS) group, n = 8.

Over the whole course of this study, administration of indoxyl sulfate and uremic toxin adsorbent AST-120 [15] had a negligible effect on body mass and food intake (data not shown). Intraperitoneal administration of indoxyl sulfate resulted in blood accumulation (p < 0.05, Figure 3A) and distribution to the prefrontal cortical tissues (p < 0.05, Figure 3B) and CSF (p < 0.05, Figure 3C), and the elevated levels were diminished by AST-120 (p < 0.05, Figure 3). Regarding behavioral changes, the Open Field Test revealed no marked alteration in locomotor activity, i.e., there was no significant difference in travel distance between the experimental and control mice (p > 0.05, Figures 4A, 4B). Intriguingly, parameters of time spent in the central zone (p < 0.05, Figure 4C) and numbers of central zone entries (p < 0.05, Figure 4D) were decreased in indoxyl sulfate-treated mice and the decreases were reversed by AST-120 (p < 0.05, Figures 4C, 4D). Furthermore, the decreased light-box preference in the Light-Dark Box Test (p < 0.05, Figure 4E) and the increased immobility time in the FST (p < 0.05, Figure 4F) and TST (p < 0.05, Figure 4G) indicated the development of anxiety-like and depression-like behaviors in the indoxyl sulfate-treated mice. AST-120 alleviated these behavioral changes (p < 0.05, Figures 4E–4G). The depression-like behavior was further demonstrated by administering an antidepressant, imipramine, in the FST (p < 0.05, Figure 4F) and TST (p < 0.05, Figure 4G). The results showed that use of imipramine abolished the depression-like behavior. With respect to spatial memory and learning, the indoxyl sulfate-treated mice in the Morris Water Maze Test showed higher escape latency distance (p < 0.05, Figure 4H) and latency time (p < 0.05, Figure 4I) in the acquisition phase. In the probe tests, indoxyl sulfate-treated mice had longer tracking paths (Figure 4J), latency distance (p < 0.05, Figure 4K), and latency time (p < 0.05, Figure 4L). AST-120 alleviated cognitive impairment in indoxyl sulfate-treated mice (p < 0.05, Figures 4H–4L) AST-120 alone had a little effect on tested behaviors (p > 0.05, Supplementary Figure 1). These findings indicate that indoxyl sulfate is an active molecule in the development of anxiety-like behavior, depression-like behavior, and cognitive impairment in unilateral nephrectomized mice. Studies have suggested biochemical changes in the prefrontal cortex, hippocampus, and amygdala have crucial roles in the development of anxiety-like behavior, depression-like behavior, and cognitive impairment [26–29]. Since the accumulation of indoxyl. sulfate can be detected in the prefrontal cortical tissues and our previous reports identify depression-related biochemical changes in the prefrontal cortical tissues [26–28], this region of the mouse’s brains was used for subsequent biochemical analyses.

### Indoxyl sulfate impaired neuronal survival and neural stem cells

**Figure 5:**
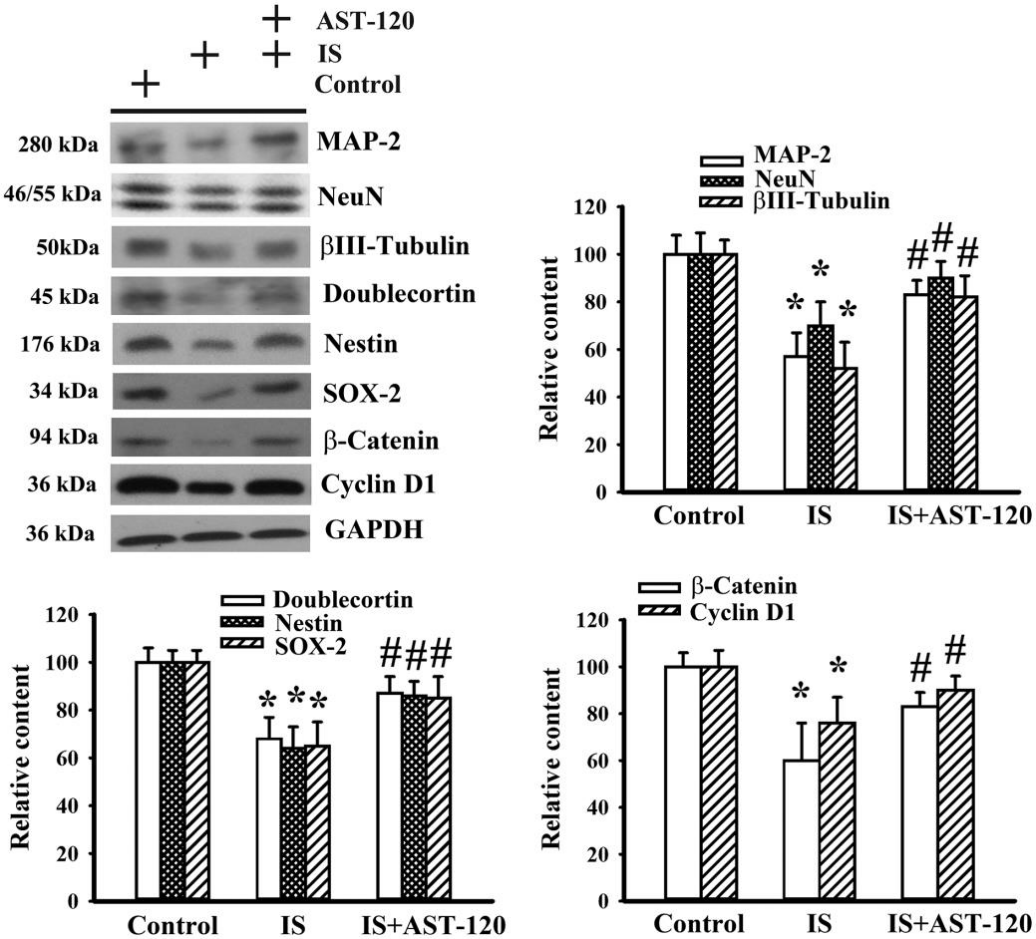
**Indoxyl sulfate decreased parameters of neuronal cell survival and neural stem cells.** Unilateral nephrectomized mice were intraperitoneally injected with indoxyl sulfate (IS, 0 and 100 mg/kg) and the indoxyl sulfate-injected mice were orally given with AST-120 (0 and 400 mg/kg) for 7 weeks. Proteins were extracted from the isolated prefrontal cortical tissues and subjected to Western blot with the indicated antibodies. Representative blots and the quantitative data are shown. *p < 0.05 vs. control group, and #p < 0.05 vs. indoxyl sulfate alone (IS) group, n = 8.

Histopathological changes in neurodegenerative diseases and depression are characterized by neuronal degeneration and impaired neurogenesis [28]. Thus, the effects of indoxyl sulfate on neuronal cell survival and neural stem cells, as well as the potential reversal effect of AST-120, were examined by Western blotting. Quantitative measurement of neuronal cell-associated Microtubule-Associated Protein 2 (MAP-2), NeuN, and β-III tubulin protein contents indicated a reduction of neuronal cells in indoxyl sulfate-treated mice (p < 0.05, Figure 5). Parallel reductions in protein contents of stem/progenitor biochemical markers of the neural lineage, including doublecortin, nestin, and SOX-2, as well as cell proliferation-associated β-catenin and cyclin D1 (p < 0.05, Figure 5) were noted. AST-120 alleviated the protein reduction in indoxyl sulfate-treated mice (p < 0.05, Figure 5). These findings indicate that indoxyl sulfate had a negative effect on neuronal cells and neural stem cell-associated genes, and that AST-120 reversed these effects.

### Indoxyl sulfate impaired neurotrophins and neurotransmitters

**Figure 6:**
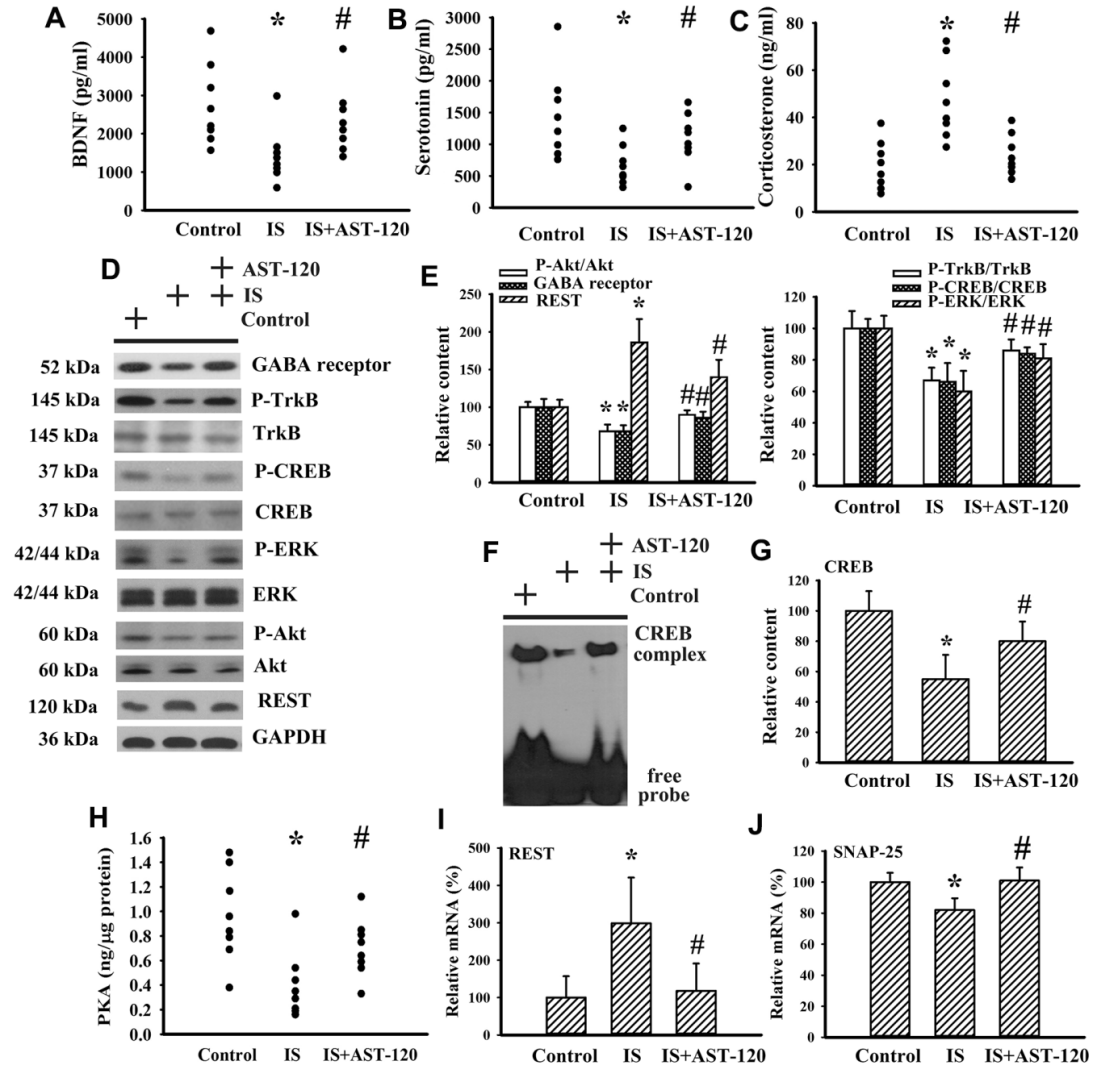
**Indoxyl sulfate decreased parameters of neurotrophins.** Unilateral nephrectomized mice were intraperitoneally injected with indoxyl sulfate (IS, 0 and 100 mg/kg) and the indoxyl sulfate-injected mice were orally given with AST-120 (0 and 400 mg/kg) for 7 weeks. The serum samples were collected and subjected to the measurement of BDNF (**A**), serotonin (**B**), and corticosterone (**C**) levels. Proteins were extracted from the isolated prefrontal cortical tissues and subjected to Western blot with the indicated antibodies. Representative blots (**D**) and the quantitative data (**E**) are shown. Nuclear proteins were extracted from the isolated prefrontal cortical tissues and subjected to EMSA for measurement of CREB DNA binding activity. Representative blots (**F**) and the quantitative data (**G**) are shown. (**H**) The prefrontal cortical tissues were isolated and subjected to the measurement of PKA activity. Total RNAs were extracted from the isolated prefrontal cortical tissues and subjected to quantitative RT-PCR for the measurement of REST (**I**) and SNAP-25 (**J**) mRNA level. *p < 0.05 vs. control group, and #p < 0.05 vs. indoxyl sulfate alone (IS) group, n = 8.

Neurotrophins, neurotransmitters, and stress hormones have crucial roles in the pathophysiology of CNS diseases, particularly through impaired Brain-Derived Neurotrophic Factor (BDNF) signaling, disturbed serotonergic neurotransmission, or overactivated corticosterone action [26–31]. Therefore, the expression levels of BDNF, serotonin, and corticosterone, and their function-associated intracellular biochemical events and molecules were examined. There was a reduction in serum level of BDNF (p < 0.05, Figure 6A) and serotonin (p < 0.05, Figure 6B), whereas the serum level of corticosterone (p < 0.05, Figure 6C) in indoxyl sulfate mice increased. The alterations in serum levels of BDNF, serotonin, and corticosterone were restored by AST-120 (p < 0.05, Figures 6A–6C). Indoxyl sulfate decreased protein expression of GABA receptor α1, protein phosphorylation of high-affinity BDNF receptor Tropomyosin-Related Kinase receptor type B (TrkB), and transcription factor cAMP Response Element-Binding Protein (CREB) (p < 0.05, Figures 6D, 6E), as well as the DNA binding activity of CREB (p < 0.05, Figures 6F, 6G). Moreover, indoxyl sulfate also had an inhibitory effect on Extracellular Signal-Regulated Kinase (ERK) phosphorylation, Akt phosphorylation (p < 0.05, Figures 6D, 6E), and Protein Kinase A (PKA) activity (p < 0.05, Figure 6H), crucial upstream activators of CREB. Downregulation of GABA receptor, TrkB, CREB, ERK, Akt (p < 0.05, Figures 6D, 6E), CREB DNA binding activity (p < 0.05, Figures 6F, 6G), and PKA (p < 0.05, Figure 6H) was abolished by AST-120. An aberrant expression of neuron-specific transcriptional repressor Repressor Element-1 Silencing Transcription Factor (REST) has been implicated in mood disorders [32]. An increased expression of REST protein (p < 0.05, Figures 6D, 6E) and mRNA (p < 0.05, Figure 6I) was found in the brains of indoxyl sulfate-treated mice and this elevation was decreased by AST-120 (p < 0.05, Figures 6D, 6E, 6I). The mRNA level of REST downstream of the Synaptosomal-Associated Protein 25 (SNAP-25) gene was decreased in indoxyl sulfate-treated mice (p < 0.05, Figure 6J). Its downregulation was alleviated by AST-120 (p < 0.05, Figure 6J). These findings suggest that indoxyl sulfate has inhibitory effects on BDNF and serotonin expression, and also induces corticosterone release. In addition, intracellular signaling and the reduction in BDNF and serotonin expression can be improved by AST-120.

### Indoxyl sulfate induced oxidative stress

**Figure 7:**
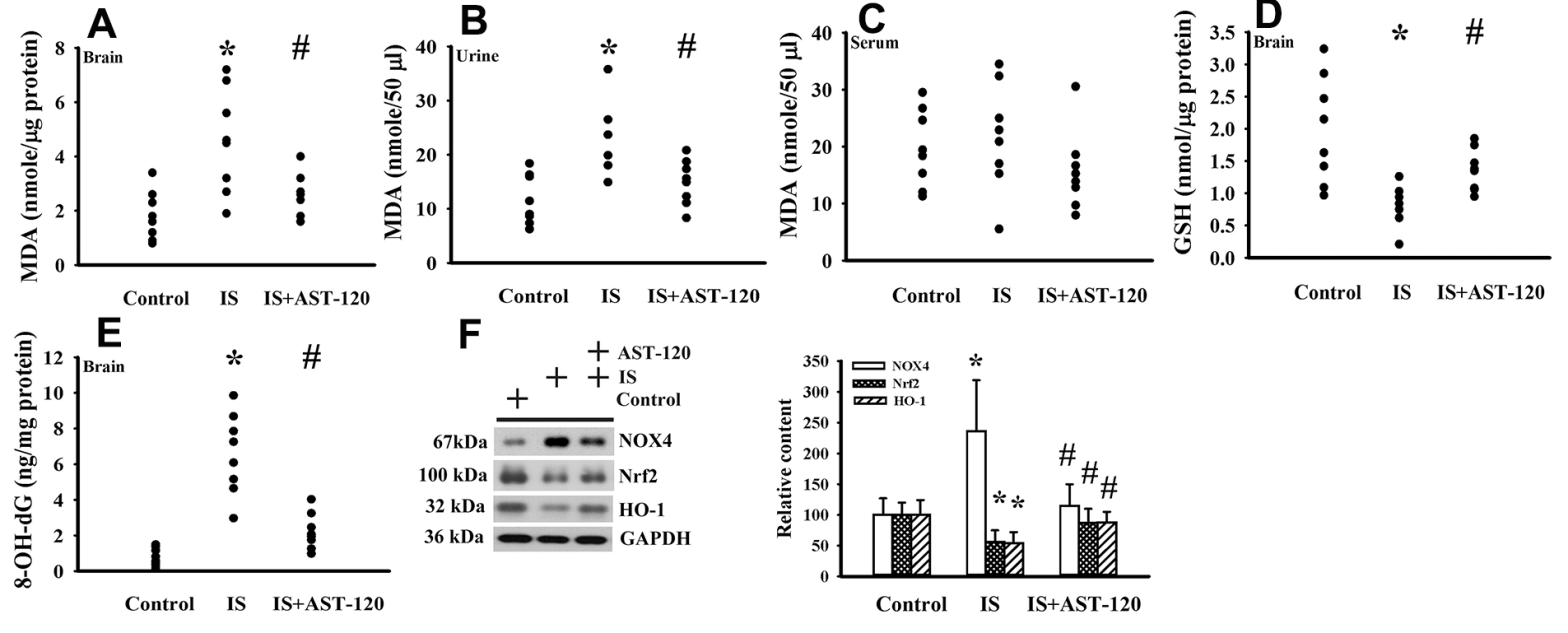
**Indoxyl sulfate induced oxidative stress.** Unilateral nephrectomized mice were intraperitoneally injected with indoxyl sulfate (IS, 0 and 100 mg/kg) and the indoxyl sulfate-injected mice were orally given with AST-120 (0 and 400 mg/kg) for 7 weeks. The prefrontal cortical tissues (**A**), urine (**B**, 24 hours), and serum (**C**) were collected and subjected to the measurement of MDA level. The prefrontal cortical tissues were collected and subjected to the measurement of GSH content (**D**). The prefrontal cortical tissues were collected and subjected to the measurement of 8-OH-dG content (**E**). Proteins were extracted from the isolated prefrontal cortical tissues and subjected to Western blot with the indicated antibodies. Representative blots and the quantitative data are shown (**F**). *p < 0.05 vs. control group, and #p < 0.05 vs. indoxyl sulfate alone (IS) group, n = 8.

**Figure 8:**
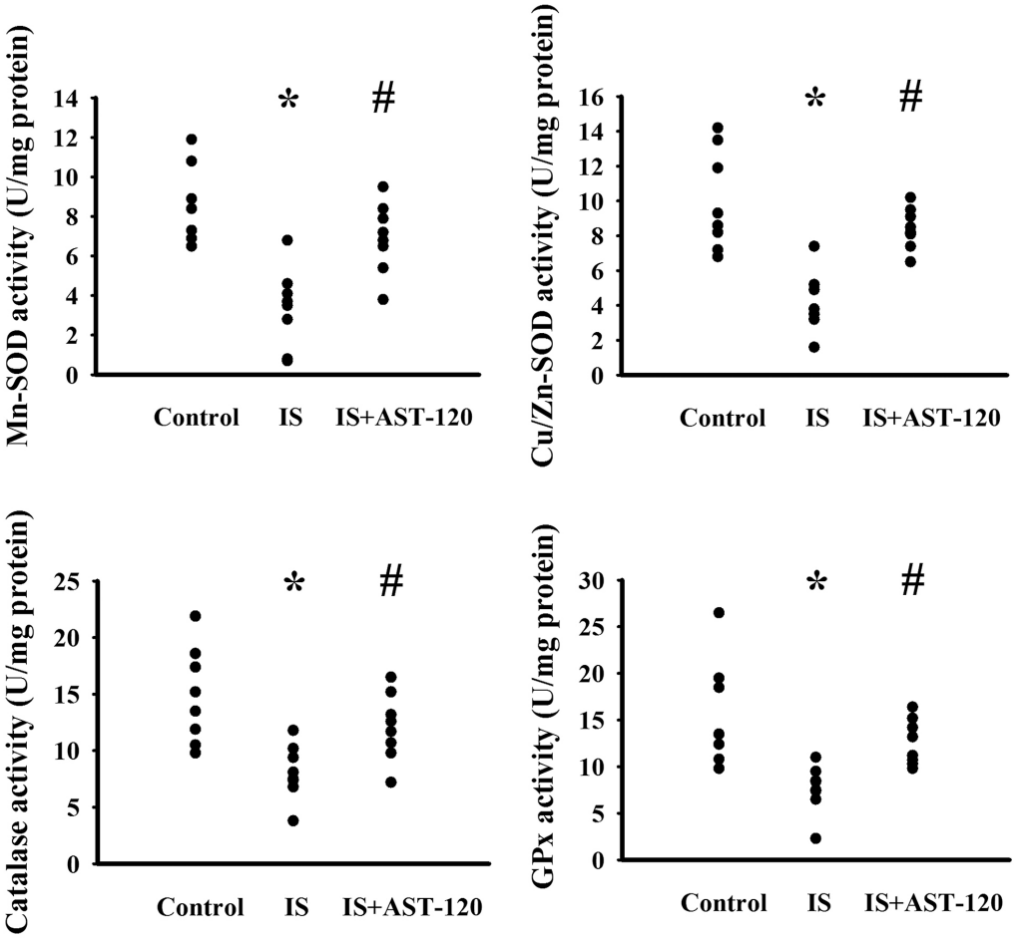
**Indoxyl sulfate decreased antioxidant enzyme activities**. Unilateral nephrectomized mice were intraperitoneally injected with indoxyl sulfate (IS, 0 and 100 mg/kg) and the indoxyl sulfate-injected mice were orally given with AST-120 (0 and 400 mg/kg) for 7 weeks. The prefrontal cortical tissues were collected and subjected to the measurement of Mn-SOD activity, Cu/Zn-SOD activity, Catalase activity, and GPx activity. *p < 0.05 vs. control group, and #p < 0.05 vs. indoxyl sulfate alone (IS) group, n = 8.

The generation of free radicals, which causes oxidative stress, has a remarkable impact on neuronal cell survival, neural stem cells, and synaptic connections, and thus has an active role in the pathogenesis of neuropsychological diseases and response to treatments [10, 26, 33, 34]. Increased levels of lipid peroxidation product Malondialdehyde (MDA) indicating oxidative stress were detected in the prefrontal cortical tissues (p < 0.05, Figure 7A) and urine (p < 0.05, Figure 7B), but not serum (p > 0.05, Figure 7C), of indoxyl sulfate-treated mice, and this effect could be reduced by AST-120 (p < 0.05, Figures 7A, 7B). The elevation of MDA level and reversal effect by AST-120 in the brain were accompanied by decreased Glutathione (GSH) content (p < 0.05, Figure 7D), increased 8-Hydroxy-2-Deoxyguanosine (8-OH-dG) content (p < 0.05, Figure 7E), elevated NADPH Oxidase 4 (NOX4) protein, and reduced NF-E2 Related Factor (Nrf2) and Heme Oxygenase-1 (HO-1) protein (p < 0.05, Figure 7F). Parallel changes were further demonstrated in Manganese-Superoxidase Dismutase (Mn-SOD) activity, Copper/Zinc-Superoxidase Dismutase (Cu/Zn-SOD) activity, Catalase activity, and Glutathione Peroxidase (GPx) activity (p < 0.05, Figure 8). These data suggest indoxyl sulfate has a pro-oxidative effect and that AST-120 can counter this effect.

### Indoxyl sulfate induced neuroinflammation

**Figure 9:**
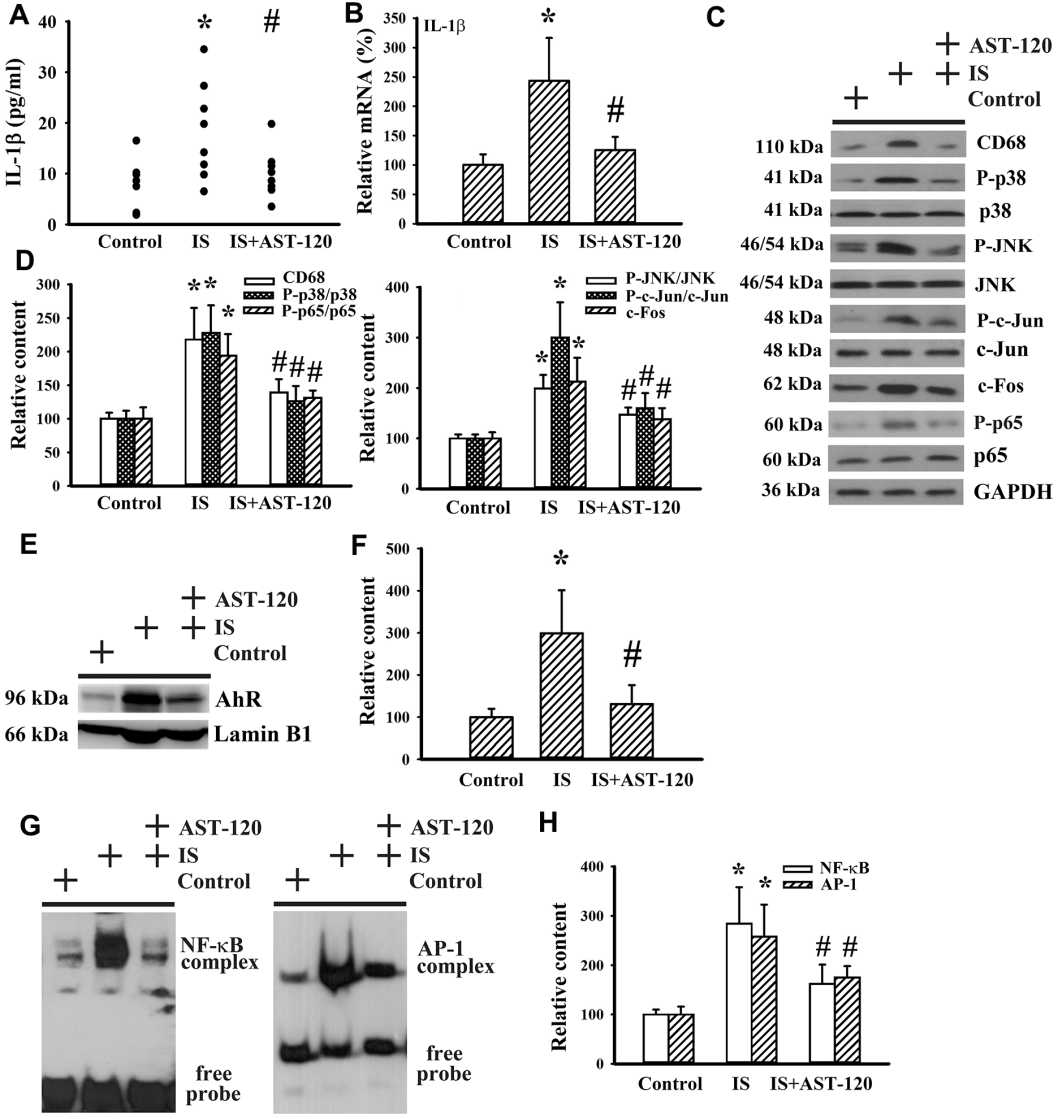
**Indoxyl sulfate induced neuroinflammation**. Unilateral nephrectomized mice were intraperitoneally injected with indoxyl sulfate (IS, 0 and 100 mg/kg) and the indoxyl sulfate-injected mice were orally given with AST-120 (0 and 400 mg/kg) for 7 weeks. (**A**) The serum samples were collected and subjected to the measurement of IL-1β level. (**B**) Total RNAs were extracted from the isolated prefrontal cortical tissues and subjected to quantitative RT-PCR for the measurement of IL-1β mRNA level. Proteins were extracted from the isolated prefrontal cortical tissues and subjected to Western blot with the indicated antibodies. Representative blots (**C**) and the quantitative data (**D**) are shown. Nuclear proteins were extracted from the isolated prefrontal cortical tissues and subjected to Western blot with the indicated antibodies. Representative blots (**E**) and the quantitative data (**F**) are shown. Nuclear proteins were extracted from the isolated prefrontal cortical tissues and subjected to EMSA for the measurement of NF- κB and AP-1 DNA binding activity. Representative blots (**G**) and the quantitative data (**H**) are shown. *p < 0.05 vs. control group, and #p < 0.05 vs. indoxyl sulfate alone (IS) group, n = 8.

Neuroinflammation has been implicated in the pathogenesis of neuropsychological diseases and anti-inflammatory treatments have been demonstrated to ameliorate disease progression [10, 33–36]. Serum level of IL-1β protein (p < 0.05, Figure 9A) and prefrontal cortical tissue level of IL-1β mRNA (p < 0.05, Figure 9B) were elevated in indoxyl sulfate-treated mice. The induction of IL-1β expression was accompanied by increased protein expression of monocyte/macrophage-associated Cluster of Differentiation 68 (CD68), Aryl Hydrocarbon Receptor (AhR), and c-Fos, protein phosphorylation of p38, c-Jun N-terminal Kinase (JNK), c-Jun, and p65 (p < 0.05, Figures 9C–9F), and DNA binding activity of NF-κB and AP-1 (p < 0.05, Figures 9G, 9H), crucial signaling molecules and transcription factors in neuroinflammation. The alterations of neuroinflammation-associated molecules were alleviated by AST-120 (p < 0.05, Figures 9C–9H). These data suggest that AST-120 improved indoxyl sulfate-induced neuroinflammation.

### Intracerebroventricular indoxyl sulfate administration caused limited alteration in behaviors

**Figure 10:**
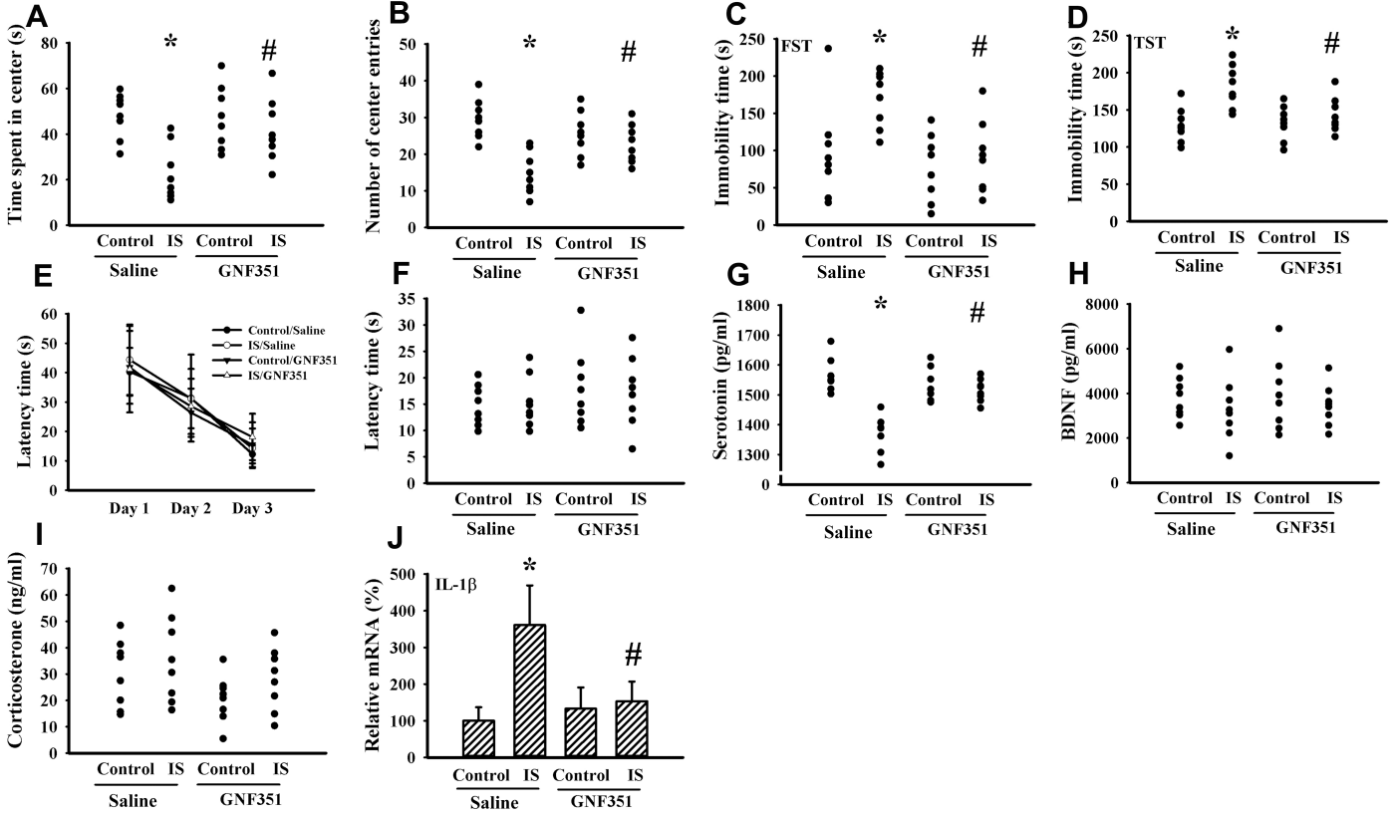
**Intracerebroventricular indoxyl sulfate administration caused behavioral alterations**. Mice were intraperitoneally injected with normal saline or GNF351 (8 mg/kg) for 30 minutes. The mice were then intracerebroventricularly injected with normal saline or indoxyl sulfate (5 μg per mouse) for 3 weeks. The time spent in the center zone (**A**) and numbers of center zone entries (**B**) were evaluated by the Open Field Test. The FST was conducted for a period of 5 min and the duration of immobility was recorded (**C**). The TST was performed for a period of 6 min and the duration of immobility was recorded (**D**). In the Morris Water Maze Test, the escape time (**E**) in the acquisition phase was recorded from 1^st^ to 3^rd^ days. After training for 3 consecutive days, the escape time (**F**) required to reach the hidden platform was recorded. The serum samples were collected and subjected to the measurement of serotonin (**G**), BDNF (**H**), and corticosterone (**I**) levels. Total RNAs were extracted from the isolated prefrontal cortical tissues and subjected to quantitative RT-PCR for the measurement of IL-1β mRNA level (**J**). *p < 0.05 vs. control saline group, and #p < 0.05 vs. indoxyl sulfate saline (IS) group, n = 8.

Indoxyl sulfate is a putative ligand for AhR and AhR antagonism mitigates inflammatory cytokine expression [37, 38]. To further elicit the CNS effects, indoxyl sulfate was administrated to normal intact mice once via intracerebroventricular infusion in the absence or presence of AhR inhibitor GNF351 [38]. Indoxyl sulfate-infused mice showed moderate alterations in the Open Field Test, FST, and TST by a decreased time spent in the central zone (p < 0.05, Figure 10A) and numbers of central zone entries (p < 0.05, Figure 10B), as well as an increased immobility time (p < 0.05, Figures 10C, 10D). Unexpectedly, escape latency time in the acquisition phase (p > 0.05, Figure 10E) and probe tests (p > 0.05, Figure 10F) was not statistically difference among groups in the Morris Water Maze Test. There was a reduction of circulating level of serotonin (p < 0.05, Figure 10G) in indoxyl sulfate-infused mice, while the levels of BDNF (p > 0.05, Figure 10H) and corticosterone (p > 0.05, Figure 10I) were not changed. Regarding neuroinflammation, an increased expression of IL-1β mRNA (p < 0.05, Figure 10J) was observed in the prefrontal cortical tissues of indoxyl sulfate-infused mice. Those alterations were alleviated by GNF351 (p < 0.05, Figures 10A, 10B, 10C, 10D, 10G, 10J). The findings suggest that indoxyl sulfate may possess some CNS effects involving AhR signaling.

## DISCUSSION

The etiologies of CNS diseases are complicated by multiple pathogenic mechanisms, including dysfunction of the hypothalamic-pituitary-adrenal axis, neurochemicals, the neuroendocrine system, neuroinflammation, oxidative stress, and neurogenesis [30, 36]. The accumulation of indoxyl sulfate in blood circulation was accelerated in unilateral nephrectomized but not the kidney intact mice after indoxyl sulfate administration at dose of 100 mg/kg/day. Herein, we further detected elevated accumulation of indoxyl sulfate in the prefrontal cortical tissues and the CSF in treated mice. Unilateral nephrectomized mice that received daily administration of indoxyl sulfate showed several behavioral signs of CNS diseases such as anxiety, depression, and cognitive impairment. However, the accumulation of indoxyl sulfate and changes of behaviors were not observed at lower doses at 1 and 10 mg/kg/day in unilateral nephrectomy nor at doses at 1, 10, and 100 mg/kg/day in intact kidneys. Of particular note was the development of depression-like behaviors, which could be reversed by the antidepressant imipramine. Those behavioral changes were accompanied by disturbed neuronal survival, neural stem cell activity, GABA receptor α1 expression, BDNF expression, serotonin expression, corticosterone expression, REST expression, and post-receptor intracellular signaling, as well as upregulated oxidative stress and neuroinflammation. Uremic toxin adsorbent AST-120 improved the above mentioned behavioral, biochemical, cellular, and molecular changes. The current findings extend our understanding of the microbiota-gut-brain axis and suggest a pathogenic role of the microbiota metabolite, indoxyl sulfate, in the development of CNS diseases involving disturbances in neural communication, neurogenesis, neuroinflammation, and oxidative stress.

The bidirectional communication between the brain and gut is mediated by the autonomic nervous system, enteric nervous system, neuroendocrine system, immune system, and gut microbiota. A growing body of evidence suggests a pathogenic role of microbes in a number of CNS diseases. The impaired drug responses and disease progression observed in patients with Parkinson’s disease are associated with Helicobacter pylori infection [39–41]. Infections with Escherichia and Shigella promote cognitive impairment and brain amyloidosis in patients with Alzheimer’s disease [42]. Studies of fecal microbiota transplantation further reveal the existence of functional pathogenic or beneficial microbes in CNS diseases [9]. Pathogenic microbes and repeated antibiotic exposure increase the disease burden of depression, while beneficial microbes and consumption of probiotics improve the disease burden of depression [7, 8, 10, 31, 33, 43]. The rebalance of gut microbiota alleviates depression features in rodent studies [18, 34, 44]. These studies highlight the pathogenic roles of disturbed gut microbes in CNS diseases involving the microbiota-gut-brain axis. Disturbed gut microbiota has a huge impact on the abundance and types of microbiota-derived molecules, including neurotransmitters, short-chain fatty acids, bile acids, and indole metabolic derivatives [45–48]. By competing with the biosynthesis of serotonin from tryptophan, intestinal microorganisms cause metabolic breakdown of tryptophan to indole derivatives via enzyme tryptophanase [46, 47]. The production of indole derivative indoxyl sulfate is elevated in disturbed gut microbiota and is decreased by probiotics [12]. Additionally, plasma levels of uremic toxins, including indoxyl sulfate, increase with age [17]. Indoxyl sulfate has been implicated in kidney and cardiovascular diseases [18], and herein, we further explored the role of indoxyl sulfate in the prevalence of CNS diseases.

The Elevated Plus Maze and the Light-Dark Box are commonly used tests for measuring the anxiety behavior of rodents. The Open Field Test is widely used for the evaluation of rodent exploration activity, locomotor activity, and anxiety. The Morris Water Maze Test and Object Recognition Test are frequently used in investigations of various aspects of memory and learning in rodents. However, depression behavior in rodents tends to be studied using the FST and TST [26–28]. In the present study, behavioral observations of unilateral nephrectomized mice subjected to the Light-Dark Box Test, FST, TST, and Morris Water Maze Test revealed that indoxyl sulfate absorption/accumulation and accompanied peripheral changes appeared to be involved in the development of anxiety-like and depression-like behaviors, as well as memory and learning deficits. As with typical pathological and molecular alterations in anxiety, depression, memory and learning impairment, neurodegeneration, impaired neurogenesis, disturbed neurotrophin and neurotransmitter signaling, oxidative stress, and neuroinflammation occurred in the brains of indoxyl sulfate-treated mice. Neurotrophins and neurotransmitters are critical regulators of neuronal homeostasis, neural plasticity, and synaptic connection. Among the various neurotrophins and neurotransmitters, BDNF and serotonin are of particular interest because they are thought to be involved in most CNS diseases [26–28]. Upon engagement of BDNF with the TrkB receptor, the BDNF/TrkB complex activates an intracellular signaling cascade via ERK, Akt, and PKA, which phosphorylates CREB and initiates transcriptional programs driving the expression of neurotrophins and neurotransmitters. In order to regulate homeostasis, REST functions as a negative regulator counterbalancing the overactivated neurotrophic actions through the transcriptional repression of neurotrophins and neurotransmitters [32]. Our findings support the results of relevant studies indicating that these biochemical events work in concert to maintain structural and functional homeostasis in the nervous system. Thus, a reduction of BDNF and serotonin signaling and/or the aberrant activation of REST, as shown in the present study following indoxyl sulfate exposure, may indicate susceptibility to neurological deficits. It should be noted that the reduction of circulating serotonin may also be a consequence of metabolic competition for tryptophan since indoxyl sulfate is capable of disrupting gut microbiota favoring the indole metabolic pathway rather than the serotonin metabolic pathway [12].

Evidence suggests that chronic intestinal inflammation, which in turn may impair the blood-brain barrier and promote neuroinflammation, neural injury, and ultimately neurodegeneration, is a potential cause of neurodegenerative conditions such as Alzheimer's and Parkinson’s disease [49, 50]. Disturbed gut microbiota is associated with oxidative stress and inflammation [12]. Indoxyl sulfate induces oxidative stress and inflammation in monocytes/macrophages and CNS glial cells [14, 37]. Upregulation of prooxidant NOX4 and downregulation of antioxidant Nrf2/HO-1 have been implicated in depression-associated oxidative stress and neuroinflammation [51]. In the brains of indoxyl sulfate-treated mice, biochemical parameters of oxidative stress and neuroinflammation were apparent when compared with those of control and AST-120-treated mice. The increased expression of monocytes/macrophages/microglia-associated CD68 protein and proinflammatory cytokine IL-1β was paralleled by upregulation of neuroinflammation-associated molecules, including p38, JNK, c-Jun, c-Fos, p65, NOX4, AP-1, and NF-κB, as well as downregulation of Nrf2 and HO-1. Since p38, JNK, c-Jun, c-Fos, p65, AP-1, and NF-κB are redox-sensitive molecules and the generation of free radicals is a consequence of an inflammatory response, the vicious cyclic biochemical cascades found in the present study could explain why indoxyl sulfate disrupts CNS integrity and causes neurological changes.

Although peripheral indoxyl sulfate administration caused its accumulation in the brains of unilateral nephrectomized mice and behavioral changes, whether CNS effects of indoxyl sulfate came from its CNS accumulation, or secondarily due to its peripheral actions was not clear. Indoxyl sulfate activates CNS glial cells and increases cytokine production [14, 15]. Upon intracerebroventricular indoxyl sulfate infusion, our data revealed that normal intact mice elevated IL-1β mRNA expression in the prefrontal cortical tissues. In parallel, the indoxyl sulfate-infused mice developed depression-like and anxiety-like behavior, while maintained cognitive function as that of control. Circulating levels of serotonin, but not BDNF and corticosterone, were moderately decreased. Despite its heterogeneous impacts, those alterations caused by indoxyl sulfate were reversed by AhR antagonism. Indoxyl sulfate has been identified as a potential endogenous ligand for AhR [37]. Evidence indicates that the AhR axis has an integrative effect in the cellular activities of immune cells and shows a pathogenic role in neurological alteration involving proinflammatory responses [52]. We found that peripheral indoxyl sulfate administration activated brain AhR in unilateral nephrectomized mice and AhR inhibitor reversed indoxyl sulfate CNS administration-induced alterations in normal mice. These findings suggest that the AhR axis could be a link between indoxyl sulfate and CNS neuroinflammation. Unlike peripheral administration in unilateral nephrectomized mice, it should be noted that the intracerebroventricular administration of indoxyl sulfate in normal mice only caused certain and limited alterations. The findings suggest that the peripheral actions of indoxyl sulfate are also pivotal to boost CNS alterations. However, the assumption is complicated by model details. Experimental parameters of indoxyl sulfate dose (5 μg per mouse), administration route (intracerebroventricular infusion), treatment course (3 weeks), and normal mice, were applied in the later model. Therefore, the exact roles of indoxyl sulfate direct CNS effects and AhR in indoxyl sulfate-induced neurological alterations should be further elucidated.

Dynamic changes in the gut microbiota have various potentially negative and positive effects on human health. Alteration in the gut microbiota has been demonstrated in patients with CKD [25]. However, bacterial generation rate in the gut is not the determinant factor for the difference in circulating uremic toxin levels between different stages of CKD [53]. Symptoms of depression, anxiety, and cognitive impairment have been reported in chronic hemodialysis patients and indoxyl sulfate was shown to be strongly associated with early-stage CKD [54, 55]. Uremic toxins are associated with worsening outcomes in CKD patients. In CKD, the gut-microbiota metabolite indoxyl sulfate progressively accumulates due to its high albumin-binding capacity, leading to clinical complications [18]. Accordingly, the pathogenic role of indoxyl sulfate in CKD-associated CNS complications is highly expected. In this study, we provide experimental evidence showing that indoxyl sulfate exerts a pathogenic effect leading to CNS diseases in unilateral nephrectomized mice. Depression-like, anxiety-like, and cognitive impairment caused by indoxyl sulfate were accompanied by indoxyl sulfate CNS accumulation, impaired neuronal cell survival and neurogenesis, disturbed BDNF, serotonin, corticosterone, and REST expression, oxidative stress, and neuroinflammation. Although current study revealed interesting findings, there were some limitations. In normal Albino Wistar rats, indoxyl sulfate (100 and 200 mg/kg) administration via drinking water for 28 days led to its increased accumulation in various brain regions and high dose of indoxyl sulfate (200 mg/kg) caused biochemical and behavioral alterations [56]. The same experimental conditions demonstrated higher plasma indoxyl sulfate concentrations in both two doses and the high dose treatment slightly impaired renal function [57]. Current study with C57BL/6 mice, an elevated circulating indoxy sulfate level was only observed in unilateral nephrectomized but not normal intact mice by daily intraperitoneal injection for 7 weeks at a dose of 100 mg/kg. Besides, there was no sign of abnormality in plasma BUN and creatinine level. The tolerance of significant renal injury was also reported by our previous studies regarding indoxyl sulfate and p-cresol sulfate [24, 58]. However, exogenous indoxyl sulfate (100 mg/kg) indeed showed peripheral effects in unilateral nephrectomized mice [24, 59]. Differences in rodent species, metabolic rate, and excretion rate may be the causes. Importantly, young but not aged mice were investigated in this study. There was one thing should be noted in the drugs administration. Uremic toxin adsorbent AST-120 was delivered by oral route, while indoxyl sulfate was administrated via intraperitoneal injection. Despite the limitations, the results presented herein still provide evidence that the protein-bound uremic toxin indoxyl sulfate may have potential as a pathogenic surrogate for CNS diseases caused, at least in part, by declined renal function. The current findings extend our understanding of the microbiota-gut-brain axis and suggest a pathogenic role of a microbiota metabolite, indoxyl sulfate, in the development of mood disorders and neurodegeneration in patients with CKD. Since indoxyl sulfate remains difficult to remove by hemodialysis, gut microbiota could be an alternative target for reducing circulating indoxyl sulfate level and its toxicity in chronic kidney disease patients, with the aim of slowing down the progression of the disease and decreasing any CNS complications.

Rodent models of severe CKD with adenine feeding or 5/6 nephrectomy show elevation of circulating indoxyl sulfate and other uremic toxins, impair renal function, and cause cognitive impairment [23]. Intraperitoneal administration of indoxyl sulfate at dose of 100 mg/kg/day for 7 weeks increased its circulating concentration from 2.30±0.58 to 7.45±2.28 mg/l in unilateral nephrectomized mice and 2.00±1.21 to 2.89±1.28 mg/l in kidney intact mice. Clinically, plasma level of indoxyl sulfate higher than 4.63 mg/l predicts progression of CKD [60]. Except indoxyl sulfate, the roles of other uremic toxins such as p-cresyl sulfate, trimethylamine, and trimethylamine N-Oxide were not addressed and documented in unilateral nephrectomized mice. Besides, only unilateral nephrectomized mice were established for current study and indoxyl sulfate was not exclusively accumulated in the prefrontal cortex. Previously, we had already reported such unilateral nephrectomized mice developed renal fibrosis despite comparable level of BUN and creatinine [59]. Despite the findings indoxyl sulfate predisposes unilateral nephrectomized mice suffering from CNS diseases, the involvement of other uremic toxins, confounding factors, gut microbiota, and effective concentrations in translating into clinical relevance requires further investigation.

## MATERIALS AND METHODS

### Study animals

The experimental protocols of nephrectomy and drug treatments in mice were reviewed and approved by the Animal Experimental Committee of Taichung Veterans General Hospital (IACUC approval code: La-1051370, IACUC approval date: Feb. 8, 2016). Ten-week-old male C57BL/6 mice (120 mice in total) were housed in a controlled animal facility for three studies. After habituation for one week, sixty eight mice were subjected to unilateral nephrectomy according to reported methods [24]. The remaining fifty two mice were kidney intact control. For the dose effect study, the intact (n = 20) and unilateral nephrectomized (n = 20) mice were allocated into four groups (n = 5 per group) receiving various doses of indoxyl sulfate (0, 1, 10, and 100 mg/kg/day) intraperitoneally for 7 weeks. The mice were used for indicated assays in Figure 1 and Figure 2. For the intervention study (Figures 3–9), forty eight nephrectomized mice were randomly allocated into three treated groups (n = 16 per group). Indoxyl sulfate (100 mg/kg) or the same volume of normal saline was intraperitoneally delivered to the mice once daily for 7 weeks. Meanwhile, spherical carbonaceous absorbent AST-120 (400 mg/kg) or normal saline was administrated to indoxyl sulfate-treated mice via a feeding tube. To minimize the confounded results due to different behavioral tasks utilized, behavioral evaluation of treated mice in the intervention study was randomly allocated into A and B groups. A group of mice (n = 8 per subgroup) was subjected to Open Field Test and Morris Water Maze Test, while the B group of mice was allocated for the evaluation of TST, FST, and Light/Dark Box Test. Controlled mice in A group were treated with imipramine for comparison. Pilot findings of FST and TST evaluation revealed that indoxyl sulfate mice showed behavioral change starting from 5 weeks after administration (data not shown). Therefore, the study course was scheduled to 7 weeks for treatments. At the end of the behavioral evaluation (7 weeks after treatment), the mice were euthanized and the prefrontal cortical tissues, blood, and urine (24 hours) were collected for further analyses. For molecular and biochemical analyses, four mice were randomly selected from A and B groups, respectively, and eight mice in total were used. For the CNS effect study, normal saline or indoxyl sulfate (5 μg per mouse) was infused through a cannula inserted perpendicularly into the coordinates: -0.9 mm posterior, 1.7 mm lateral to the sagittal suture, and 2.2 mm in depth. The intracerebroventricular infusion in the intact mice was lasted for 1 hour under anesthesia with isoflurane. Thirty minutes before intracerebroventricular infusion, mice were intraperitoneally injected with normal saline or GNF351 (8 mg/kg). The mice (n = 8 per group) were maintained for 3 weeks and used for indicated assays in Figure 10.

### Measurement of indoxyl sulfate

The prefrontal cortical tissues, serum, and CSF were isolated and subjected to the measurement of indoxyl sulfate using high-performance liquid chromatography in accordance with our reported methods [18].

### Behavioral evaluations

All behavioral tests were conducted during the light cycle phase in enclosed behavior rooms. The modified Open Field Test, FST, TST, Light-Dark Box Test, and Morris Water Maze Test were evaluated in accordance with previously reported methods by technicians blinded to the treatments [26–28]. In the Open Field Test, mice were placed on the apparatus (L x30 cm and W x30 cm) with 16 squares for a period of 30 min. Travel distance, time spent in the central zone, and numbers of central entries were measured. For the measurement of FST, and TST, mice were forced to swim for 5 min and individually suspended by the tail for 6 min, respectively, and the immobility time was recorded. To evaluate the effect of antidepressant, imipramine (20 mg/kg) was intraperitoneally administrated 1 h prior to the tests. For the Light-Dark Box Test, mice were placed in the apparatus (L x40 cm, W x30 cm, and H x30 cm) for a period of 5 min. The light and dark areas each constituted half of the available area within the box. In each experiment, the mouse was placed into the illuminated part of the box and the time that it remained there was recorded. In the Morris Water Maze Test, the evaluation was performed in an illuminated room with some external signs that remained in the same place during probe tests and training. The experimental apparatus consisted of a circular pool with a diameter of 150 cm at a depth of 60 cm. The water (25° C) was rendered opaque with powdered milk. The surface of the pool was virtually divided by four quadrants and the escape platform (10 cm in diameter) was located 1 cm below the water surface near the center of one quadrant of the pool. Mice received three acquisition trials per day for three consecutive days and the training trials were separated with 1 minute intertrial intervals. Mice were placed randomly in one of the quadrants and a maximum 90 seconds was allowed to find and climb the hidden platform. If mice failed to find the platform within 90 seconds, they were guided to the escape platform. Parameters of escape latency distance and latency time were measured. An average was calculated for three daily trials. On day 4, the time required to reach the hidden platform and the total distance traveled by the mouse were recorded. All swimming trials were recorded with a video camera placed on the ceiling and analyzed using Etho Vision 3.1 (Noldus, The Netherlands) software.

### Western blot

The prefrontal cortical tissues were isolated and proteins were extracted using tissue protein extraction reagents (T-PER, Pierce Biotechnology, Rockford, IL, USA). Equal amounts of proteins were electrophoretically separated and transferred to blotting membranes. The membranes were sequentially incubated with primary antibodies and horseradish peroxidase-conjugated secondary antibodies followed by enhanced chemiluminescence visualization. The immunoreactive bands were quantified by a densitometer. The antibodies used were against MAP-2 (1:1000, sc-74421), nestin (1:1000, sc-23927), Glyceraldehyde-3-Phosphate Dehydrogenase (1:5000, GAPDH, sc-47724), ERK (1:2000, sc-514302), phosphorylated ERK (1:2000, sc-7383), SOX-2 (1:1000, sc-365823), cyclin D1 (1:2000, sc-8396), doublecortin (1:1000, sc-271390), TrkB (1:2000, sc-8316), CD68 (1:1000, sc-20060), Akt (1:2000, sc-8312), phosphorylated Akt (1:2000, sc-271966), p38 (1:2000, sc-7972), phosphorylated p38 (1:2000, sc-17852-R), GABA receptor α1 (1:1000, sc-37682), JNK (1:1000, sc-7345), phosphorylated JNK (1:1000, sc-6254), c-Jun (1:1000, sc-74543), phosphorylated c-Jun (1:1000, sc-16312), c-Fos (1:1000, sc-52), p65 (1:1000, sc-372), phosphorylated p65 (1:1000, sc-136548), β-catenin (1:1000, sc-7963), AhR (1:2000, sc-398877), lamin B1 (1:1000, sc-374015), REST (1:1000, sc-374661) (Santa Cruz Biotechnology, Santa Cruz, CA, USA), NeuN (1:1000, ab128886), phosphorylated TrkB (1:500, ab131483), NOX4 (1:1000, ab109225), Nrf2 (1:1000, ab137550), HO-1 (1:500, ab13248) (Abcam, Cambridge, UK), βIII-tubulin (1:3000, AB9354, Millipore, Billerica, MA, USA), CREB (1:1000, #9104), and phosphorylated CREB (1:1000, #9191) (Cell Signaling, Danvers, MA, USA).

### RNA isolation and quantitative real-time reverse transcriptase polymerase chain reaction (RT-PCR)

The prefrontal cortical tissues were isolated and following extraction of total RNA, cDNA was synthesized according to previously reported methods [27]. The expression of mRNA was measured by quantitative real-time PCR using ABI StepOne^™^ (Applied Biosystems, Foster City, CA, USA) and its level was calculated by the ΔΔCT method normalized with β-actin. Oligonucleotide sequences used in PCR were 5′-TCAGGCAGGCAGTATCACTC and 5′-AGCTCATATGGGTCCGACAG for IL-1 β; 5’-ACATGCGTAATGAACTGGAG and 5’-GA|GCAAGGCGAACAACTGGAACG for SNAP-25; 5′-CCTGCAGCAAGTGCAACTAC and 5′-CTTCTGAGAGCTTGAGTAAGG for REST; 5′-CACGATGGAGGGGCCGGACTCATC and 5′-TAAAGACCTCTATGCCAACACAGT for β -actin.

### Measurement of BDNF, serotonin, IL-1β, and corticosterone content

The serum samples were isolated and subjected to the measurement of BDNF, serotonin, IL-1β, and corticosterone using the corresponding Enzyme-Linked Immunosorbent Assay (ELISA) kits, according to the manufacturer’s instructions (R&D Systems, Minneapolis, MN, USA).

### Measurement of PKA activity

The prefrontal cortical tissues were isolated and subjected to PKA activity measurement using a commercially available PKA kinase activity assay kit (Enzo Life Sciences, NY, USA), according to the manufacturer’s instructions. The calculated activity was expressed as amounts of active PKA protein (ng/μg protein).

### Preparation of nuclear extracts and electrophoretic mobility shift assay (EMSA)

The prefrontal cortical tissues were isolated and subjected to extraction of nuclear proteins using a commercial assay kit (NE-PER Nuclear and Cytoplasmic Extraction Kit, ThermoFisher Scientific, IL, USA). An EMSA assay kit (LightShift^™^ Chemiluminescent EMSA Kit, ThermoFisher Scientific, IL, USA) was used for the measurement of DNA binding activity of NF-κB, AP-1, and CREB, according to the manufacturer’s instructions. The reactive DNA/protein complexes were detected using chemiluminescence reagents. The oligonucleotides recognizing NF-κB, AP-1, and CREB were 5’-AGTTGAGGGGACTTTCCCAGGC, 5’-CGCTTGATGAGTCAGCCGGAA, and 5’-AGAGATTGCCTGACGTCAGAGAGCTAG, respectively.

### Measurement of lipid peroxidation

The prefrontal cortical tissues, serum, and urine were isolated and subjected to measurement of lipid peroxidation products using a Thiobarbituric Acid Reactive Substances (TBARS) assay kit (ZeptoMetrix, Buffalo, NY, USA). The calculated levels of lipid peroxidation products were expressed as MDA equivalents, according to the manufacturer’s instructions.

### Measurement of antioxidant enzyme activity

The prefrontal cortical tissues were collected and isolated. The levels of reduced GSH and activities of Mn-SOD, Cu/Zn-SOD, Catalase, and GPx were measured using commercially available assay kits (Cayman, Ann Arbor, MI, USA).

### Measurement of 8-OH-dG

The prefrontal cortical tissues were collected and isolated. The levels of 8-OH-dG were measured using commercially available assay kits (8-hydroxy 2 deoxyguanosine ELISA Kit, ab201734, Cayman, Ann Arbor, MI, USA).

### Serum biochemical measurement

The serum levels of BUN and creatinine were measured using Automated Clinical Chemistry Analyzer (FUJIFILM DRI-CHEM 4000i, Tokyo, Japan).

### Statistical analysis

The data are expressed as mean values ± standard deviation. Statistical analysis was performed using One-Way or Two-Way Analysis of Variance, followed by Dunnett’s test or Tukey’s test for post hoc tests through all experiments. A level of p < 0.05 was considered statistically significant.

## Supplementary Material

Supplementary Figure 1
