# Supplementary material for: Indoxyl sulfate caused behavioral abnormality and neurodegeneration in mice with unilateral nephrectomy
Source: Aging (Albany NY). 2021 Feb 17;13(5):6681–701. doi: 10.18632/aging.202523 (PMC7993681; doi:10.18632/aging.202523)
Supplement: Supplementary Figure 1 [file aging-13-202523-s001.pdf]

SUPPLEMENTARY FIGURES

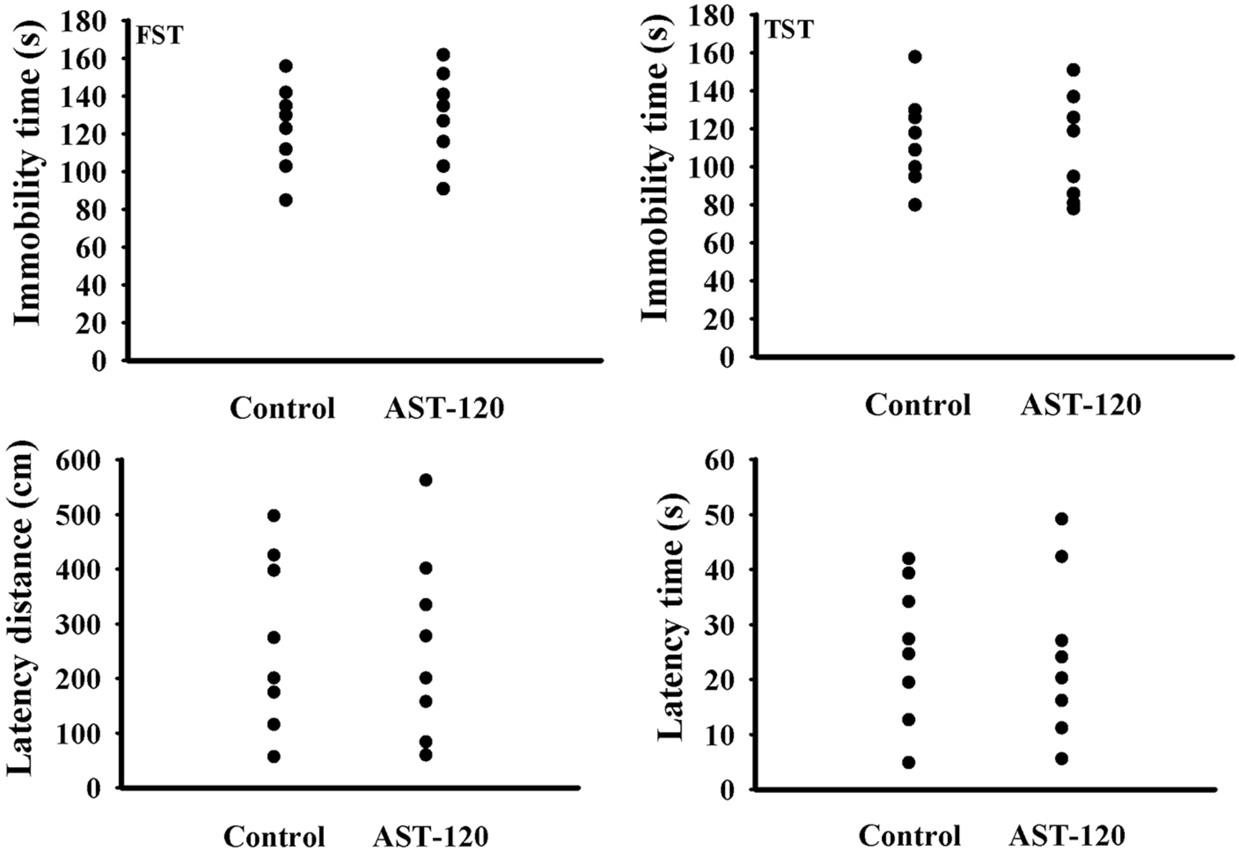

Supplementary Figure 1. AST-120 alone had a little effect on behaviors. Unilateral nephrectomized mice were orally given with AST-120 (0 and 400 mg/kg) for 7 weeks. The FST was conducted for a period of 5 min and the duration of immobility was recorded. The TST was performed for a period of 6 min and the duration of immobility was recorded. After training for 3 consecutive days, the escape distance and escape time required to reach the hidden platform were recorded in the Morris Water Maze Test. N = 8.
